# Coherent Interpretation of Entire Visual Field Test Reports Using a Multimodal Large Language Model (ChatGPT)

**DOI:** 10.3390/vision9020033

**Published:** 2025-04-11

**Authors:** Jeremy C. K. Tan

**Affiliations:** 1 Faculty of Medicine, University of New South Wales, Kensington, NSW 2033, Australia; jeremy.c.tan@unsw.edu.au; 2 Save Sight Institute, University of Sydney, Sydney, NSW 2000, Australia; 3 Prince of Wales Hospital Eye Clinic, High Street, Level 4, The Prince of Wales Hospital, High Street Building, Randwick, NSW 2031, Australia

**Keywords:** large language model, vision language model, glaucoma, visual field

## Abstract

This study assesses the accuracy and consistency of a commercially available large language model (LLM) in extracting and interpreting sensitivity and reliability data from entire visual field (VF) test reports for the evaluation of glaucomatous defects. Single-page anonymised VF test reports from 60 eyes of 60 subjects were analysed by an LLM (ChatGPT 4o) across four domains—test reliability, defect type, defect severity and overall diagnosis. The main outcome measures were accuracy of data extraction, interpretation of glaucomatous field defects and diagnostic classification. The LLM displayed 100% accuracy in the extraction of global sensitivity and reliability metrics and in classifying test reliability. It also demonstrated high accuracy (96.7%) in diagnosing whether the VF defect was consistent with a healthy, suspect or glaucomatous eye. The accuracy in correctly defining the type of defect was moderate (73.3%), which only partially improved when provided with a more defined region of interest. The causes of incorrect defect type were mostly attributed to the wrong location, particularly confusing the superior and inferior hemifields. Numerical/text-based data extraction and interpretation was overall notably superior to image-based interpretation of VF defects. This study demonstrates the potential and also limitations of multimodal LLMs in processing multimodal medical investigation data such as VF reports.

## 1. Introduction

Glaucoma is a leading cause of blindness worldwide, and the visual field (VF) test is an integral tool in both its diagnosis and management [1]. A threshold VF test report typically contains a number of summary and location-specific metrics that describe the sensitivity and reliability properties of the test, such as the mean deviation (MD) and pointwise threshold sensitivity. These metrics are assessed by the clinician to determine if a glaucomatous VF defect is present or if the VF has progressed compared to previous tests, in combination with other modalities such as structural data and the intraocular pressure. The process of VF interpretation can, however, be highly subjective, and while multiple validated scoring systems exist [2], they are often complex and not suited for routine clinical practice. Unaided clinical judgement is consequently inconsistent, with interobserver agreement among even expert observers being moderate at best [3,4,5]. Artificial intelligence (AI)-assisted systems may improve the accuracy and consistency and shorten the clinician’s time required for interpretation [6]. This is increasingly critical, particularly in hospital-based eye clinics given the high patient turnover and resource-intensive nature of ophthalmology clinics [7].

Recently, LLMs have been equipped with expanded capabilities for analysis of user-uploaded images and their potential uses in medicine has been described [8]. The multimodal capabilities of emerging multimodal LLMs may therefore be useful in extracting pertinent data from raw imaging reports and combined with clinical data for interpretation. For instance, we recently demonstrated the use of LLMs in the interpretation of biometry reports for cataract surgery pre-operative planning [9]. This study evaluates the accuracy and consistency of a general, publicly available LLM in interpreting VF test reports for the assessment of glaucoma.

## 2. Methods

This was a retrospective study conducted at the Prince of Wales Hospital Department of Ophthalmology, a tertiary referral eye unit in Sydney, Australia. Ethics approval for this study was provided by the South Eastern Sydney Local Health District institutional ethics review board (2024/ETH02244). VF data were extracted from a secure database of anonymised test reports previously described [10,11]. The VFs included in this analysis were threshold 24-2 tests performed with the Humphrey Field Analyzer (HFA, Carl Zeiss, Meditec, Dublin, CA, USA) and the Swedish Interactive Testing Algorithm (SITA) strategy. A representative set of 60 VF tests of 60 patients comprising the three currently available SITA strategies—SITA-Standard, -Fast and -Faster—across healthy, suspect or manifest glaucoma and across varying severities based on the mean deviation (MD) was used. The latter was classified into mild (MD > −6 dB), moderate (MD > −12 and ≤ −6 dB) and severe (MD ≤ −12 dB) glaucoma. Tests of poor reliability based on established thresholds from the standard reliability indices [false positives (FPs) or fixation losses (FLs)] were also deliberately included. The following parameters were first extracted from the VF reports using a custom Python (version 3.13.2) script containing a pdf reader for numerical/string characters of global sensitivity [MD, pattern standard deviation (PSD), and Glaucoma Hemifield Test (GHT)] and reliability (FL and FP) indices and an optical character recognition algorithm for the pattern deviation (PD) probability grid scores. The outputs from this script were verified to ensure full accuracy in data extraction. If a defect was present on the PD grid, the type(s) of defect was then recorded by the author (e.g., superior arcuate defect).

### 2.1. Analysis of Raw Imaging Data by LLM

**Figure 1:**
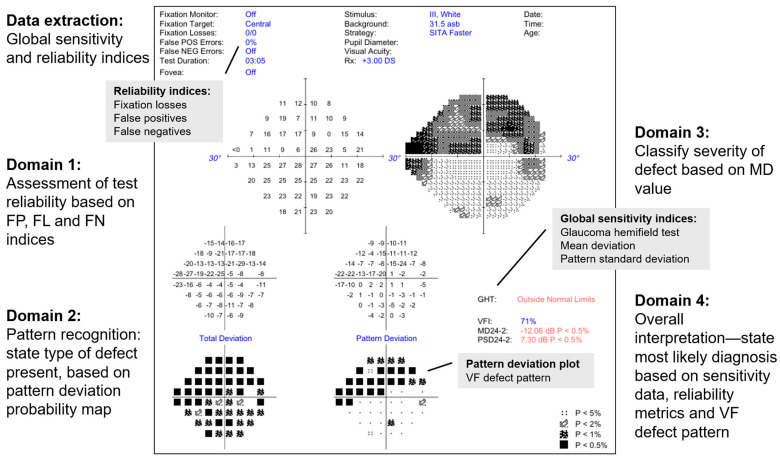
Entire single-field VF reports were uploaded to the LLM. System prompts were used to extract the global sensitivity and reliability indices from the reports. The model was then assessed on its ability to assess and interpret the VF test report across four domains—test reliability, defect type based on the pattern deviation probability grid, defect severity based on mean deviation value and overall diagnosis based on all the above information.

The anonymised single-page VF test reports were then uploaded as image files into a commercially available LLM (GPT-4o, OpenAI, San Francisco, CA, USA). The web-based chat interface was used, as the Vision API was not currently accessible directly via OpenAI’s public API at the time of writing. System prompts were used to extract the sensitivity and reliability indices from the reports. The model was then assessed on its ability to assess and interpret the VF test report across four domains—test reliability, defect type, defect severity and overall diagnosis. The system prompt accompanying each uploaded image included four questions to achieve this (Figure 1):

1. Is this a reliable VF test? (Reliability)
  - The model is expected to consider FP, FL and false negative values to judge if the test reliability is acceptable for interpretation
2. What type of VF defect is present? (Type of defect)
  - This is based on the PD grid (e.g., nasal step defect)
3. If this patient has glaucoma, what is the severity of the disease based on this defect? (Severity)
  - The model is expected to classify the severity (mild, moderate or severe) based on the MD value.
4. What is the most likely diagnosis based on this VF test alone? (Diagnosis)
  - The model is expected to consider the global sensitivity and reliability indices and what type of VF defect is present, to provide a verdict as to whether the VF test result is consistent with healthy, suspect or manifest glaucoma.

While the diagnosis of glaucoma is usually made in conjunction with structural information (optic disc appearance and retinal nerve fibre thinning on optical coherence tomography), the model was asked to use VF information alone to provide the most *likely* diagnosis.

### 2.2. Assessment of Model Outputs

**Figure 2:**
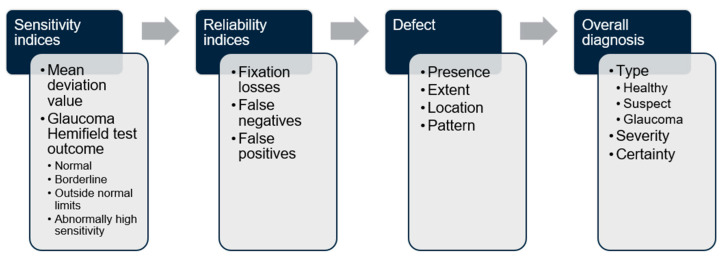
Flow chart of the reasoning process expected to answer the question as to what the overall diagnosis of the VF test report is. This relies on the integration of sensitivity indices (MD and GHT outcome), reliability indices (low/acceptable base FL, FP and FN) and defect characteristics to provide an overall diagnosis along with qualification of the severity and certainty of the latter.

The extracted metrics were first verified against outputs from the custom script for accuracy. Domain 1 was then assessed by comparison with the widely established 15% FP or 20% FL thresholds, although the former threshold has been observed to be potentially too strict for SITA-Faster in recent publications [1,12]. Domain 2 was assessed based on several levels of descriptions in an increasingly specific order from 1 to 4: (1) defect presence/absence: was a defect on the total/pattern deviation grid detected; (2) defect extent: were all components of the defect described; (3) defect location: was the defect localised to the correct superior/inferior and nasal/temporal hemifield/quadrant; and (4) defect pattern: was the pattern of defect [13] (e.g., arcuate, nasal step) correctly described. The latter was at times found to be non-specific [13]; therefore, in the absence of a clear pattern that fit with the retinal ganglion cell axon arrangement (e.g., nasal step), non-specific responses were not considered incorrect. If a clear pattern was observed yet the LLM provided an obviously different pattern, this was then only considered incorrect. Likewise, if the LLM made a clear error in defining a characteristic in the other three levels (e.g., misstating a superior defect as inferior, or an inferonasal defect as superior), this was only then considered incorrect. Domain 3 was a simple classification task based on the extracted MD value. Domain 4 was a higher-level reasoning task based on the integration of sensitivity indices (MD and GHT outcome), reliability indices (low/acceptable base FL, FP and FN) and defect characteristics to provide an overall diagnosis along with qualification of the severity and certainty of the latter (Figure 2).

## 3. Results

### 3.1. Baseline Characteristics of Cohort

VF test reports from 60 eyes of 60 patients were included, with a mean age of 65.0 (SD 14.6) years and baseline MD of −5.52 (SD 6.5) dB (Table 1). In total, 16 reports were originally flagged by the instrument as having low reliability due to elevated false positives or fixation losses.

### 3.2. Performance Across Domains of VF Analysis

**Figure 3:**
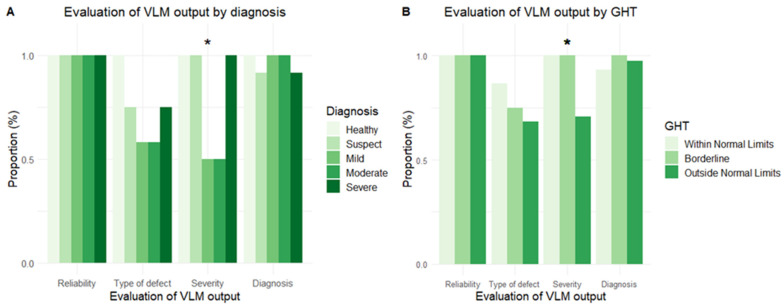
Proportion of correct responses for four domains (1: test reliability, 2: type of defect, 3: defect severity and 4: overall diagnosis) of VF interpretation across diagnoses (**A**) and glaucoma hemifield test results (**B**). Moderate performance was observed for domains 2 and 3, with significant differences in C noted across diagnoses and GHT results. GHT: glaucoma hemifield test, LLM: vision language model. The asterisk denotes a significant difference in proportion across diagnosis and GHT subcategories in classifying the severity of the VF defect.

The LLM displayed perfect (100%) accuracy in extracting global sensitivity (MD, PSD, VFI and GHT) and reliability (FP, FN and FL) metrics. The accuracy of the LLM in the analysis and interpretation of glaucomatous field defects was thereafter assessed via four domains, as previously described. The LLM displayed perfect accuracy in domain 1—judging the reliability of the VF test—and near-perfect (96.7%) accuracy in domain 4—interpreting whether the VF defect was consistent with glaucoma with consideration of all the sensitivity and reliability metrics. The accuracy in correctly defining the type of (domain 2) and severity (domain 3) of the defect was, however, only 73.3% and 80.0%, respectively. There was a significant difference across diagnoses in the accuracy of classifying the severity of the defect (χ^2^ = 22.5, *p* < 0.001), with errors made only in cases of mild and moderate glaucoma. Errors in defining the type of defect were observed in suspect and all three severity levels of manifest glaucoma, though this difference across diagnoses was not significant (Figure 3). There was likewise a significant difference across GHT levels in the accuracy of classifying the severity of the defect (χ^2^ = 6.95, *p* = 0.03), but no significant difference in the other three domains.

### 3.3. Test–Retest Performance and Investigating Errors in Defect Type

A subset of VF reports (n = 15) was fed back into the LLM to assess the consistency of responses across the four domains. In this cohort, the accuracy across the domains was 100% in domain 1, 66.7% in 2 and 86.7% in 3 and 4, respectively. On retesting, the accuracy in domain 1 remained as 100%, while the accuracy in both domains 3 and 4 improved to 100%. The performance of domain 2, however, worsened to 60.0%.

**Figure 4:**
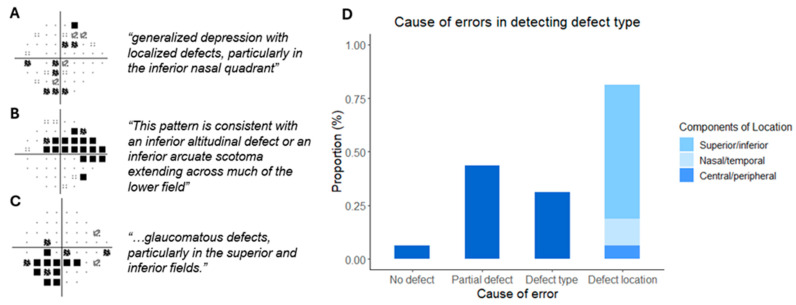
Causes of errors in defining the type of defect present. 3 examples of incorrect LLM outputs for the corresponding pattern deviation probability grid defect on the left. All three grids are of the left eye (**A**–**C**). Causes of errors for defining the type of defect, which comprised not detecting the defect completely, detecting only part of the defect, stating the wrong type of defect and stating the incorrect location of the defect. The breakdown for incorrect location is also shown (**D**).

The reduced performance in defining the type of defect (domain 2, n = 16 eyes) was interrogated further. The most common cause of error was describing the defect in the wrong location (81.3%), followed by only describing part of the defect (43.8%), defining the wrong type of defect (31.3%) and not picking up the defect at all in one instance. For defects defined in the wrong location, the most common error was confusing the superior/inferior orientation (62.5%), followed by the nasal/temporal orientation and central/peripheral location (Figure 4). For VF reports where an error of defect type was made, the region of interest showing the pattern deviation map alone was cropped and fed back into the LLM to assess if the accuracy of the output could be improved. Here, the LLM was able to correctly define the type of defect in 7 (43.8%) of the 16 eyes. Once again, the location of the defect was the most common cause of error (n = 7), followed by incomplete defect (n = 4) and wrong defect type (n = 3).

### 3.4. VF Report Interpretation in Cases of Suspected Glaucoma

The VF reports for cases of suspected glaucoma provided an opportunity for the reasoning capabilities of the LLM to be assessed in more detail. This is because of the discordance between sensitivity outputs and defect characteristics (e.g., normal MD and borderline GHT, but with a defect present) that may be observed in glaucoma suspect eyes. In this cohort, the accuracy for domains 1 and 3 was 100%, while the defect type was incorrectly described in three cases. The LLM provided two possible diagnoses in four reports (three labelled healthy or suspected glaucoma and one labelled suspected or early glaucoma). A wrong diagnosis label (healthy) was given in one eye, which had a central defect on the pattern deviation grid despite a normal MD and GHT outcome. The LLM qualified its output diagnosis by flagging the GHT outcome in eight eyes, the defect in six eyes and reliability in four eyes, indicating the reliance on GHT outcome for the interpretation (Table 2).

## 4. Discussion

In this study, the accuracy and consistency of an LLM in interpreting entire VF test reports for the evaluation of glaucoma was assessed. The LLM displayed not only the ability to extract the summary indices of interest with perfect accuracy, but an almost fully accurate performance in judging the reliability of the test, whether the defect was consistent with a healthy, suspect or glaucomatous eye, and the disease severity on retesting. The LLM’s performance was, however, notably reduced when asked to define the type of defect present, which only improved partially when provided with a more defined region of interest.

### 4.1. LLM in the Interpretation of VF Test Reports

The misclassification of severity likely reflects inconsistent classification using the MD value, as the LLM was able to define the standard, established thresholds it used to define severity. Given the marked improvement of this on retesting, self-refinement of outputs could be potentially used by re-inserting the initial output back into the model to improve accuracy. Conversely, the modest performance in defining the type of defect is likely due to the model’s algorithmic limitations in classifying defects. Upon interrogation, the LLM was found to use a convolutional neural network (CNN) trained on a labelled dataset of VF images to classify defects. The accuracy of CNN-based classification can vary significantly depending on the training dataset [14,15]. For instance, a study by Kucur et al. reported an average precision of 88% for classification of early-glaucoma VFs from controls [16]. A model trained specifically on VF data may therefore provide improved classification of defects. Furthermore, the lack of a standardised consensus system for VF classification [2] also complicates how defects are described. Nevertheless, the model made clear errors in confusing the orientation of defects in relation to the test grid and at times failed to detect part or the entirety of the defect. This may suggest a more fundamental limitation in the model’s computer vision capabilities, which was only partially improved by narrowing the region of interest to the PD grid. Akgun et al. likewise evaluated ChatGPT-4 in interpreting 30 VF printouts with or without defects [17]. While the authors found a high model accuracy in identifying test names, reliability and global VF indices, interpretation of total deviation and greyscale maps was only 66.7% and 30%, respectively [17]. These findings indicate that while extraction and interpretation of numerical/text-based information were highly accurate, the interpretation of images or patterns remains inconsistent/poor. It could be that a CNN-based classification system for VF defects may not provide high enough levels of accuracy—a potential solution instead may be a rule-based algorithm based on clearly defined patterns from a consensus classification system.

In this study, the LLM displayed high accuracy in providing the correct diagnosis in all except 2 of the 60 eyes. The LLM’s output in describing the diagnosis reflected its ability to integrate sensitivity and reliability indices and characteristics of the defect in making this interpretation. In addition, the LLM provided reasons for the diagnosis given in all cases, and even recommended repeat testing in cases of low reliability. Akgun et al., however, reported that the LLM provided an accurate diagnosis in only one-third of the 30 cases evaluated [17]. This could, however, be in part from how the diagnosis of glaucoma is specifically defined and the difficulty in providing an accurate diagnosis based on VF information alone (refer to limitations).

### 4.2. Integrating LLM into Clinical Workflow

This study underscores the potential of the LLM in healthcare given its excellent performance in extracting and synthesising data from raw diagnostic imaging reports, which may assist in the detection of pathology and also support clinician productivity. This may be particularly useful in specialities like ophthalmology with high patient volumes and which rank among the busiest outpatient departments given the nature of follow-up required for chronic conditions [7]. Analogous to AI applications in radiology, which assist clinicians in detecting abnormalities and generating preliminary reports [18,19], LLMs could similarly support clinicians through its multimodal vision capabilities, which integrate text-based and image data with reasoning abilities. Potential applications may include its use as an educational tool [20], the automated assessment of report quality and the summarisation of key findings for the clinician. For instance, visual field reports could be analysed by an LLM to screen for poor quality and provide a preliminary interpretation before being read by the clinician. This may be of particular value in low-resourced settings where the availability of trained personnel familiar with the diagnosis of glaucoma is often limited. Given the only moderate accuracy in characterising defects as shown in this study, an LLM may benefit from integration with a dedicated rule-based computer vision system, or undergo further training on specific datasets (e.g., a large quantity of VF data). A multimodal approach that combines LLMs with outputs from image- and text-based clinical data to create decision support tools could potentially improve clinic workflow and productivity [21], though these systems will need frameworks to ensure data security and patient privacy/confidentiality [22]. Further prospective studies are required to evaluate the implementation of these systems in clinical workflow, to assess issues such as physician trust, model interpretability and medicolegal considerations and to determine whether these models can be seamlessly integrated into routine clinical decision-making.

### 4.3. Limitations

Only VF reports from one type of instrument were used. Given that the HFA is one of the most commonly used instruments in VF testing, the LLM may be less familiar with the interpretation of reports from other types of instruments, depending on what type of data the model has been trained upon. Further studies evaluating the performance of the LLM on reports from other instruments may be of interest. This study was also restricted to the use of VF reports in healthy eyes and glaucoma. It may, however, be useful to evaluate the model’s performance on VF reports containing neurological defects and its ability to distinguish these from glaucomatous defects. For instance, Nikdel et al. evaluated the performance of ChatGPT-4 in interpreting Hess charts and automated VF reports in cases of neurological lesions, and found variable responses. Studies on larger datasets of VF reports with neurological abnormalities may be required to evaluate this further.

Furthermore, as mentioned earlier the diagnosis of glaucoma is usually made in conjunction with structural information (optic disc appearance and retinal nerve fibre thinning on optical coherence tomography), especially in early/suspected glaucoma where defects may be non-repeatable. The model was, however, specifically asked to use VF information alone to provide the most *likely* diagnosis. Future studies could examine whether the LLM can integrate accompanying structural information (optical coherence tomography reports) to provide a more holistic diagnosis [23,24,25]. Finally, this study did not analyse which components of the extracted data were associated with correct/incorrect predictions, which would have enhanced model explainability.

## 5. Conclusions

A commercially available multimodal large language model was able to extract summary indices from entire single-page VF reports with perfect accuracy, perform image-processing of defects and diagnose the presence or absence of potentially glaucomatous changes. Numerical/text-based data extraction and interpretation was overall notably superior to image-based interpretation of VF defects. This demonstrates the potential and limitations of multimodal LLMs in supporting ophthalmic healthcare workflow.

## Figures and Tables

**Table 1:** Baseline demographic and ophthalmic characteristics of cohort (n = 60 eyes) from which VF test reports were obtained. Standard deviation of continuous variables is shown in brackets. The Glaucoma cohort contained equal numbers of mild, moderate and severe glaucoma. SITA: Swedish interactive thresholding algorithm, MD: mean deviation, PSD: pattern standard deviation, GHT: glaucoma hemifield test, FP: false positive.

	Diagnosis		
	Healthy	Suspect	Glaucoma
Number	12	12	36
Age	64.5 (17.1)	62.0 (9.5)	66.1 (15.3)
Eye, right	8	4	18
SITA Strategy			
Standard	4	4	12
Fast	4	4	12
Faster	4	4	12
MD	0.03 (1.0)	−0.53 (0.9)	−9.0 (6.2)
PSD	1.6 (0.3)	2.2 (0.5)	8.1 (3.6)
GHT			
Within normal limits	12	2	0
Borderline	0	4	0
Outside normal limits	0	6	36
FP	5.3 (4.8)	13.2 (16.0)	4.3 (6.4)
FP > 15%	2	6	8

**Table 2:** Sensitivity and reliability indices, defect presence/absence and model outputs for the 12 VF reports labelled as glaucoma suspects.

**Number**	Sensitivity Indices			Reliability Indices			Defect	Model Output		
	MD	PSD	GHT	FL	FN (%)	FP (%)		Diagnosis	Severity	Certainty
1	0.59	2.21	Within Normal Limits	12	0	12	Present	Healthy	NA	Nil
2	−0.33	2.88	Outside Normal Limits	2	0	0	Present	Early/suspect	mild	Flagged GHT, defect
3	−0.43	2.58	Borderline	4	6	3	Present	Suspect	mild	Flagged GHT, reliability
4	−1.31	2.3	Outside Normal Limits	4	8	18	Present	Suspect	mild	Flagged GHT, defect, reliability
5	−0.74	1.72	Borderline	0	0	3	Present	Suspect	mild	Flagged GHT, defect
6	−0.01	1.96	Borderline	0	0	0	Present	Suspect	mild	Flagged GHT, defect
7	−0.41	2.75	Outside Normal Limits	0	NA	30	Present	Suspect	mild	Flagged GHT, defect
8	−0.71	2.72	Outside Normal Limits	0	0	37	Present	Suspect	mild	Flagged GHT, defect
9	−0.43	1.42	Within Normal Limits	0	0	0	Nil	Healthy	NA	Nil
10	0.82	2.75	Outside Normal Limits	5	17	46	Present	Healthy/suspect	NA	Flagged reliability
11	−2.8	1.68	Outside Normal Limits	0	0	0	Non-specific	Healthy/suspect	NA	Nil
12	−0.63	1.86	Borderline	0	11	7	Present	Healthy/suspect	NA	Flagged GHT, reliability

## Data Availability

Data are contained within the article.

## Abbreviations

Large language model (LLM), vision language model (VLM), visual field (VF), mean deviation (MD), artificial intelligence (AI), Swedish interactive thresholding algorithm (SITA), convolutional neural network (CNN), pattern deviation (PD).
